# A model study of terraced riverbeds as novel ecosystems

**DOI:** 10.1038/s41598-020-60706-y

**Published:** 2020-03-02

**Authors:** Hezi Yizhaq, Moshe Shachak, Ehud Meron

**Affiliations:** 1 Department of Solar Energy and Environmental Physics, Blaustein Institutes for Desert Research, Ben-Gurion University of the Negev, Sede Boqer Campus, Midreshet Ben Gurion, 8499000 Israel; 2 Mitrani Department of Desert Ecology, Blaustein Institutes for Desert Research, Ben-Gurion University of the Negev, Sede Boqer Campus, Midreshet Ben Gurion, 8499000 Israel; 3 Physics Department, Ben-Gurion University of the Negev, Beer Sheva, 8410501 Israel

**Keywords:** Community ecology, Community ecology, Ecological modelling, Ecological modelling

## Abstract

Riverbed terracing has been introduced in ancient times to retain water and soil, to reduce hydrological connectivity and erosion and to increase primary and secondary productivity of agro-ecological systems. These presently abandoned human-made landscapes have become novel ecosystems and a potential source of ecosystem services to humans in drylands. We use a mathematical-modeling approach to study factors that regulate terraced riverbeds and affect community and ecosystem attributes such as productivity, functional diversity and resilience to droughts. We introduce a model that captures the relationships between rainfall pattern, runoff coupling between adjacent terraces, and vegetation growth, taking into account competition for water and light. We found that a large number of weak rainfall events results in lower total biomass and functional diversity across the terraced riverbed compared with a few strong rainfall events. We further analyzed the filtering of species traits from pools of functional groups that make different tradeoffs between investment in above-ground biomass to capture canopy resources and investment in below-ground biomass to capture soil resources. Pools characterized by concave tradeoffs give rise to higher functional diversity, lower biomass production and lower resilience to droughts, as compared with convex pools. New empirical studies are needed to test these model predictions.

## Introduction

**Figure 1:**
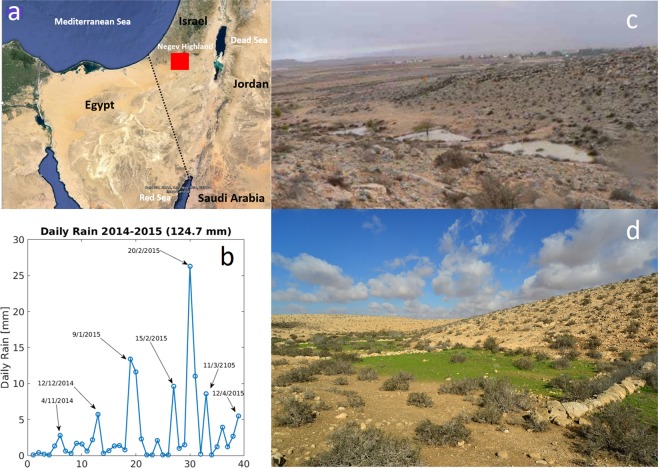
Terraced riverbeds in the Negev Highland. (**a**) The red rectangle indicates the area of the Negev Highland, where most riverbeds are terraced. (**b**) Rainfall events in Sde Boker located in the Negev highland during the winter of 2014–2015. (**c**) Old terraces in the Negev Highland from the Roman–Byzantine-era still capture runoff water during heavy rain events. (**d**) During the winter and spring, the terraces are covered with a carpet of annuals which are still used today for grazing by the local Bedouin.

Ancient terraces, covering large areas of dry riverbeds, are striking hallmarks of systematic human intervention in arid landscapes. Riverbed terracing has been introduced as a means to retain water, stabilize soil, reduce hydrological connectivity and erosion^1–4^, and increase primary and secondary productivity of agro-ecological systems (Fig. 1). Constructed of stones that were collected from their surroundings, ancient terraces were used to grow cereals, such as wheat and barley, in the Middle Eastern drylands, and orchards of fruit trees, such as olives, almonds and vines, in more humid regions^5–7^.

In spite of their large extent as present legacy of human cultural landscape, most of the research concerned with terraces focused on their structure and function in relation to past cultures. Only recently has the scientific community acknowledged the importance of terraces as a human-made integrated hydro-geo-ecosystem that can provide a bundle of ecosystem services to humans in drylands, at present time and in the future^7,8^.

Runoff harvesting, soil conservation and soil cultivation ceased in ancient terraced riverbeds as a result of significant demographic, social and economic changes. These changes have resulted in networks of landscapes consisting of novel ecosystems within abandoned ancient farming terraces^5^. Rehabilitation of degraded drylands aiming at increasing ecological productivity and diversity should view the network of the abandoned terraces as an ecological legacy where the impacts of past landscape modifications regulate current ecological functions^9^. This legacy, the outcome of terrace construction, cultivation and abandonment, can be regarded as comprising hydrological, geomorphological and ecosystem legacies, as described below.

The hydrological legacy is associated with the natural network of water-flow pathways that has been modified by the construction of stone-wall terraces. The terraces imped riverbed water runoff and create soil-moisture enriched areas within the terraces^10^; during runoff-generating rainfall events the terraces capture the water flow that would otherwise run off the watershed^11^. The hydrological legacy is therefore reflected by reducing water connectivity and leakage while increasing soil moisture on the watershed level.

The geomorphological legacy is related to the modification of the dry-riverbed topography and soil properties. The stepped micro-topography of the terraced fields decreases the energy of the overland flow and significantly reduces the erosion of soil, as compared with natural valleys. These functional aspects of terraced riverbeds have gradually disappeared when farming practices were abandoned, and the natural buildup of drainage networks has increased soil erosion and transport of sediment by runoff. These processes are especially visible in semi-arid environments with sparse plant cover that were subjected to irregular rainfall events and long periods of drought. The net effect of the soil erosion processes is a significant reduction in soil quality and its function in C and N cycling.

The negative effects of terrace abandonment are partially compensated by natural ecological processes that form the present ecological legacy of the terraced system. Abandonment is followed by re-establishment of the dry valley through natural vegetation that grows on the watershed slopes. Annual and perennial plants species and their associated animal communities create new ecosystems in conjunction with the abiotic conditions of the terraces dictated by the hydrological and geomorphological legacies, termed novel ecosystems^6^. The self-organized ecosystem offers an increase of water retention and decrease in soil erosion mainly by woody plants that function as hydrological engineers and enhance the soil infiltration capacity. In addition, herbaceous and woody plant species improved soil structure and function due to increased organic content resulting from litter fall and decomposition. Thus, abandonment of farming practices, followed by the re-colonization of natural vegetation, has improved soil functions in relation to water and nutrients fluxes, and created effective protection against soil erosion^12,13^.

Terraces, along with their hydro-geo-eco legacies, are crucial components of the ecological landscape of drylands, but very little is known about the soil-vegetation feedbacks underlying hydro-geo-eco processes of terraced areas and their effect on productivity and diversity. Despite their long periods of abandonment, ancient terraces constitute a reusable landscape capital for combating desertification and mitigation of climate change by enabling local retention of water and organic matter and sustaining plant communities^8^. Restoring their function, re-designing their water-connectivity architecture, and re-managing them in accord with rainfall variability, can result in productive and resilient dryland ecosystems.

Assessing the potential of ancient terraces as a landscape capital can highly benefit from mathematical modeling and model studies. Models of dryland ecosystems^14–16^ have already proven to be instrumental in understanding self-organized vegetation patchiness^17–19^ and functional diversity of plant communities^20^. The aim of this paper is twofold. We first use a meta-ecosystem modeling approach^21–23^, and earlier dryland-ecosystem models^24^, to introduce a new terraced-riverbed model that captures the relationships between rainfall pattern, runoff coupling between adjacent terraces, and the differential plant-community dynamics in the terraces. We then use the model to study whole riverbed responses, in terms of biomass production and functional diversity, to different rainfall regimes, varying the tradeoff form that characterizes the species pool. The specific model we study is motivated by the structure of the extensive terraced system in the Negev desert (Fig. 1), wherein an area of about 2000 km^2^, terraced riverbeds extend to a total length of about 1000 km^25^. However, the model can be used to study other riverbed systems by using different parameter values or modifying terms in the model equations.

## A Meta-Ecosystem Model for Terraced Riverbeds

**Figure 2:**
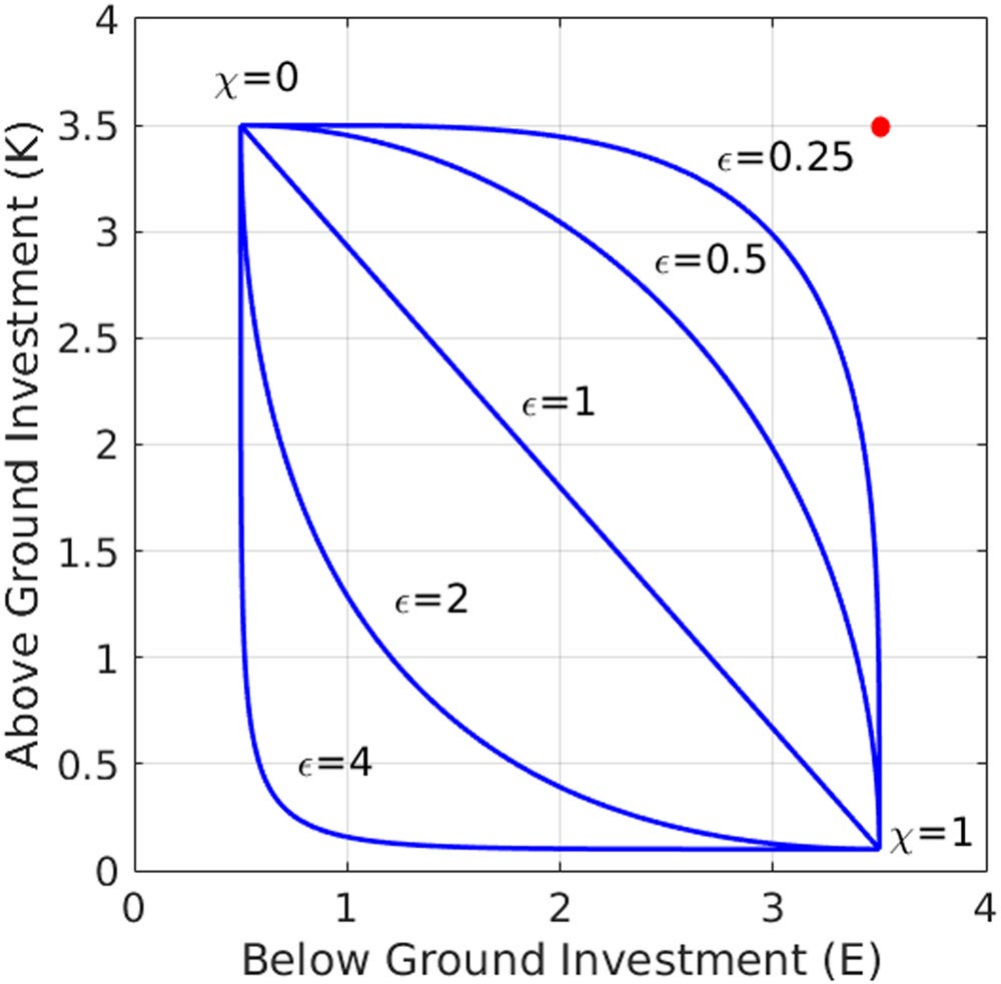
Different tradeoff curves obtained from equations (1): linear tradeoff $({\epsilon }=1)$, convex tradeoff $({\epsilon } < 1)$, and concave tradeoff $({\epsilon } > 1)$. The red circle denotes an “ideal functional group” $({E}_{max},\,{K}_{max})$ not restricted by a tradeoff. Convex tradeoffs represent pools with competitive advantage given to functional groups with intermediate χ values (closer to the red circle), while concave tradeoffs represent advantage to groups with extreme χ values, either small or large.

Our starting point is a model for a single terrace (ecosystem) that describes a water-limited plant community^24,26^. It consists of two water variables, below-ground or soil water, $W$, and above-ground or surface water, $H$, and biomass variables ${B}_{1},{B}_{2},\ldots ,{B}_{m}$, representing the above-ground biomass of $m$ functional groups. We focus on functional groups of species that make different compromises in their relative investments in above-ground biomass (shoot), to capture canopy resources, and below-ground biomass (root), to capture soil resources. Specifically, we focus on two functional traits, the maximal shoot biomass $k$, and the root-to-shoot ratio $E$, and introduce a dimensionless tradeoff parameter $\chi \in [0,1]$, defined implicitly through the relations:

1a $$K(\chi )={K}_{min}+{(1-\chi )}^{{\epsilon }}({K}_{max}-{K}_{min}),$$

1b $$E(\chi )={E}_{min}+{\chi }^{{\epsilon }}({E}_{max}-{E}_{min}),$$

where ${K}_{min}$, ${K}_{max}$ and ${E}_{min}$, ${E}_{max}$ determine the ranges of the two traits in the pool of functional groups, and ${\epsilon }$ is a positive constant (Nathan *et al*., 2016). Thus, $\chi =0$ represents a functional group that invests mostly in shoots to capture light, $(K,E)=({K}_{max},\,{E}_{min})$, while $\chi =1$ represents a functional group that invests mostly in roots to capture soil water, $(K,E)=({K}_{min},\,{E}_{max})$. The distributions of all other functional groups ${\chi }_{i}$, in between these two extremal groups, strongly depend on the value of ${\epsilon }$ as the tradeoff curves in Fig. 2 show.

The significance of ${\epsilon }$ can be understood by considering an ideal functional group of species $({K}_{max},\,{E}_{max})$, a “Darwinian demon”^27^, that benefits from high values of both $K$ and $E$ (red point in Fig. 2). Functional groups that are closer to this ideal group are expected to have competitive advantage over other groups. The closest functional group can be calculated by minimizing the distance function

1c $$s(\chi )=\sqrt{{[K(\chi )-{K}_{max}]}^{2}+{[E(\chi )-{E}_{max}]}^{2}},$$

with respect to $\chi$, where $K(\chi )$ and $E(\chi )$ are given by $(1a)$ and $(1b)$. For ${\epsilon }=1$ (linear tradeoff), such a calculation gives the intermediate value

1d $${\chi }_{mid}=\frac{{({E}_{max}-{E}_{min})}^{2}}{{({E}_{max}-{E}_{min})}^{2}+{({K}_{max}-{K}_{min})}^{2}},$$

for the closest, and thus most competitive, functional group. As ${\epsilon }$ is decreased below unity (convex tradeoff) a distinguished set of functional groups around ${\chi }_{mid}$ becomes significantly closer to the ideal group $({K}_{max},\,{E}_{max})$ than all other groups. By contrast, as ${\epsilon }$ is increased beyond unity (concave tradeoff) the distance to the ideal functional group becomes more uniform across the pool, implying a wider range of functional groups with nearly equal competitive capabilities. Note that for sufficiently high ${\epsilon }$ values the shortest distance to the ideal group occurs at the two extreme groups, $\chi =0$ and $\chi =1$.

The biomass variable associated with the *i*th functional group is ${B}_{i}=B({\chi }_{i})$, that is the biomass obtained by solving the model equations with the particular parameter values ${K}_{i}=K({\chi }_{i})$ and ${E}_{i}=E({\chi }_{i})$, keeping all other parameters fixed. For simplicity, we assume that in each terrace all water and biomass variables are spatially uniform. This assumption holds for homogeneous systems with weak pattern-forming feedbacks^19,28^.

The terraced riverbed (meta-ecosystem) to be studied here is obtained by coupling the single-terrace models for adjacent terraces through the above-ground water variable to account for runoff contributions from upslope terraces and loses to downslope terraces. Note that we did not include sediment transport from upslope terraces as this is a secondary effect. The meta-ecosystem model for a riverbed with $n$ terraces reads

2a $$\frac{d{B}_{ij}}{dt}=\frac{{\varLambda }_{ij}{W}_{j}}{1+a{W}_{j}}{(1+{E}_{i}{B}_{ij})}^{2}(1-\frac{{B}_{ij}}{{K}_{i}}){B}_{ij}-{M}_{i}{B}_{ij},$$

2b $$\frac{d{W}_{j}}{dt}=I{H}_{j}-L{W}_{j}-\frac{{W}_{j}}{1+a{W}_{j}}\mathop{\sum }\limits_{i=1}^{m}{\varGamma }_{i}{B}_{ij}{(1+{E}_{i}{B}_{ij})}^{2},$$

2c $$\frac{d{H}_{j}}{dt}=P-I{H}_{j}+D({H}_{j+1}-{H}_{j}),\,j\le n-1$$

2d $$\frac{d{H}_{n}}{dt}=(1+\alpha )P-I{H}_{n}-D{H}_{n},$$

where the index $i\,\,$runs over the $m$ functional groups, the index $\,j\,\,$runs over the $n$ terraces, ${B}_{ij}$ is the above-ground biomass of the *i*^th^ functional group in the *j*^th^ terrace, and ${W}_{j}$ and ${H}_{j}$ represent, respectively, the contents of soil water and of surface water in the *j*^th^ terrace. The factor ${\Lambda }_{ij}$ in equation $(1a)$ is given by:

3 $${\Lambda }_{ij}={\Lambda }_{0}(1-\frac{{B}_{Tj}-{B}_{ij}}{{B}_{Tj}+{B}_{R}}),{B}_{Tj}=\mathop{\sum }\limits_{i=1}^{m}{B}_{ij},$$

and represents growth attenuation of species that suffer from competition for light (Nathan *et al*., 2016). This competition is strongly affected by the maximal-shoot biomass parameters, ${K}_{i}$, which affect the late-stage growth of the plant species. These parameters represent species-specific growth limitation, assumed here as resulting from self-shading, and thus related to specific leaf area. Competition for water is captured by the water-dependent biomass growth factors ${(1+{E}_{i}{B}_{ij})}^{2}{W}_{j}/(1+a{W}_{j})$ in Eq. (1a) and by the water-uptake rates $\sum _{i}{\varGamma }_{i}{B}_{ij}{(1+{E}_{i}{B}_{ij})}^{2}$ in Eq. (1b). That competition is strongly affected by the root-to-shoot ratios, ${E}_{i}$, of the different functional groups. The source of soil water ${W}_{j}$ in any terrace comes from the infiltration of surface water ${H}_{j}$ at a rate $I$. The source of surface water comes from precipitation at a time-dependent rate $P$ and runoff contributions from adjacent upper terraces and from surrounding hillsides. The latter contribution often comes predominantly from the hillslopes that surround the uppermost terrace $j=n$ and is modeled in Eq. (2d) by the term $\alpha P$, where $\alpha$ is a function of the precipitation rate given by

4 $$\alpha =\frac{{\alpha }_{1}{P}^{2}}{1+{\alpha }_{2}{P}^{2}}.$$

**Figure 3:**
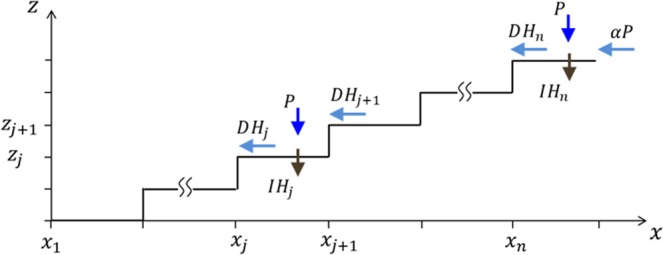
An illustration of surface-water dynamics in terraced riverbed. Gain of surface water in the *j*th terrace is due to precipitation, $P$, and runoff contribution, $D{H}_{j+1}$, from the adjacent upper terrace. Loss of surface water in the *j*th terrace is caused by infiltration into the soil, $I{H}_{j}$, and by runoff, $D{H}_{j}$, to the adjacent lower terrace. The uppermost terrace receives a runoff contribution, $\alpha P$, from the surrounding hillslopes.

This form captures the diminishingly small runoff contribution at low $P$ when the soil is dry, $\alpha P\approx {\alpha }_{1}{P}^{3}\ll P$, the surge in that contribution as $P$ increases, and its attenuation at high $P$, as the soil becomes saturated and the runoff-contribution approaches a linear dependence, $\alpha P\approx (\frac{{\alpha }_{1}}{{\alpha }_{2}})P$, with a proportionality factor $\frac{{\alpha }_{1}}{{\alpha }_{2}}$ that reflects the watershed. The runoff coupling between adjacent terraces is introduced through the terms $D({H}_{j+1}-{H}_{j})$ in Eq. (2c), where the parameter $D$ provides a measure for the coupling strength. An illustration of the factors that affect surface-water dynamics in a terraced riverbed is shown in Fig. 3.

The reader is referred to Table 1 for a list of all model parameters, their meanings, units and numerical values. A few more comments are in order. We assumed that the biomass loss parameter ${M}_{i}$, which represents mortality and additional factors such as grazing, is uniform across the community ${M}_{i}=M$. This is unlike an earlier study^20^ where a distinction between short and long grazing histories was made. We have not modeled transport of soil water between the terraces as it is much slower that the transport of surface water^8^. We have not included an evaporation term in the surface-water Eq. (2c,d) because of the fast conversion of surface water to soil water through infiltration. For simplicity, the runoff rate $D$ is considered to be a constant. In practice, runoff is slowed down by vegetation and $D$ is biomass dependent. We note that $D$ is related to the uniform extension $l={x}_{j+1}-{x}_{j}$ of the terraces and to their uniform height $d={z}_{j+1}-{z}_{j}$ (see Fig. 3) through $\propto \frac{d}{{l}^{2}}$.

**Table 1:** A list of dimensional parameters appearing in the model equations. The values shown in the table are based to a large extent on an earlier study (Nathan *et al*., 2016), and are used throughout the simulations, unless specifically stated otherwise. Values in square brackets show the minimal and maximal values of the parameter range that was studied.

Parameter	Meaning	Value	Units
$T$	Time	variable	${\rm{yr}}$
${\Lambda }_{0}$	Growth rate of unit biomass density per unit water available	0.3	${{\rm{m}}}^{2}/({\rm{kg}}\cdot {\rm{y}})$
${B}_{R}$	Reference value for above ground biomass, above which competition for light is significant	20.0	${\rm{kg}}/{{\rm{m}}}^{2}$
$m$	Number of functional groups	500	$-$
$n$	Number of terraces	9	$-$
$A$	Soil-water areal density at which the water-uptake rate is half of the maximal water-uptake rate	60	${\rm{kg}}/{{\rm{m}}}^{2}$
${K}_{i},\,{K}_{min},\,{K}_{max}$	Actual, minimal, and maximal values of the standing biomass limit, representing growth constraints such as self-shading	variable, 0.1, 3.5	${\rm{kg}}/{{\rm{m}}}^{2}$
${E}_{i},\,{E}_{min},\,{E}_{max}$	Actual, minimal and maximal values measuring root-to-shoot ratio	variable, 0.5, 3.5	${{\rm{m}}}^{2}/{\rm{kg}}$
${M}_{i}=M$	Biomass loss rate in dry soil assumed equal for all functional groups	0.1	${{\rm{y}}}^{-1}$
$D$	Runoff rate between adjacent terraces	0.64	${{\rm{y}}}^{-1}$
${\varGamma }_{i}=\varGamma$	Water uptake rate assumed equal for all functional groups	1.0	${{\rm{m}}}^{2}/({\rm{kg}}\cdot {\rm{y}})$
$L$	Evaporation rate of soil water	2.5	${{\rm{y}}}^{-1}$
$I$	Infiltration rate of soil water	5.0	${{\rm{y}}}^{-1}$
$P$	Precipitation rate	[0,100]	${\rm{mm}}/{\rm{y}}$
${\alpha }_{1,}{\alpha }_{2}$	Parameters that characterize the asymptotic forms of the runoff-contribution function $\alpha (P)$ at low and high $P$ values (Eq. 4).	0.0156, 0.0052 respectively	${{\rm{y}}}^{2}/{{\rm{mm}}}^{2}$
$k$	Number of rainfall event in a year	variable	$-$
$T$	Duration of a rainfall event	1	$day$
$A$	Intensity of a rainfall event	variable	${\rm{mm}}/{\rm{day}}$
$MAP=kAT$	Mean annual rainfall	100	mm/day

## Results

### Biomass distributions along the tradeoff axis

**Figure 4:**
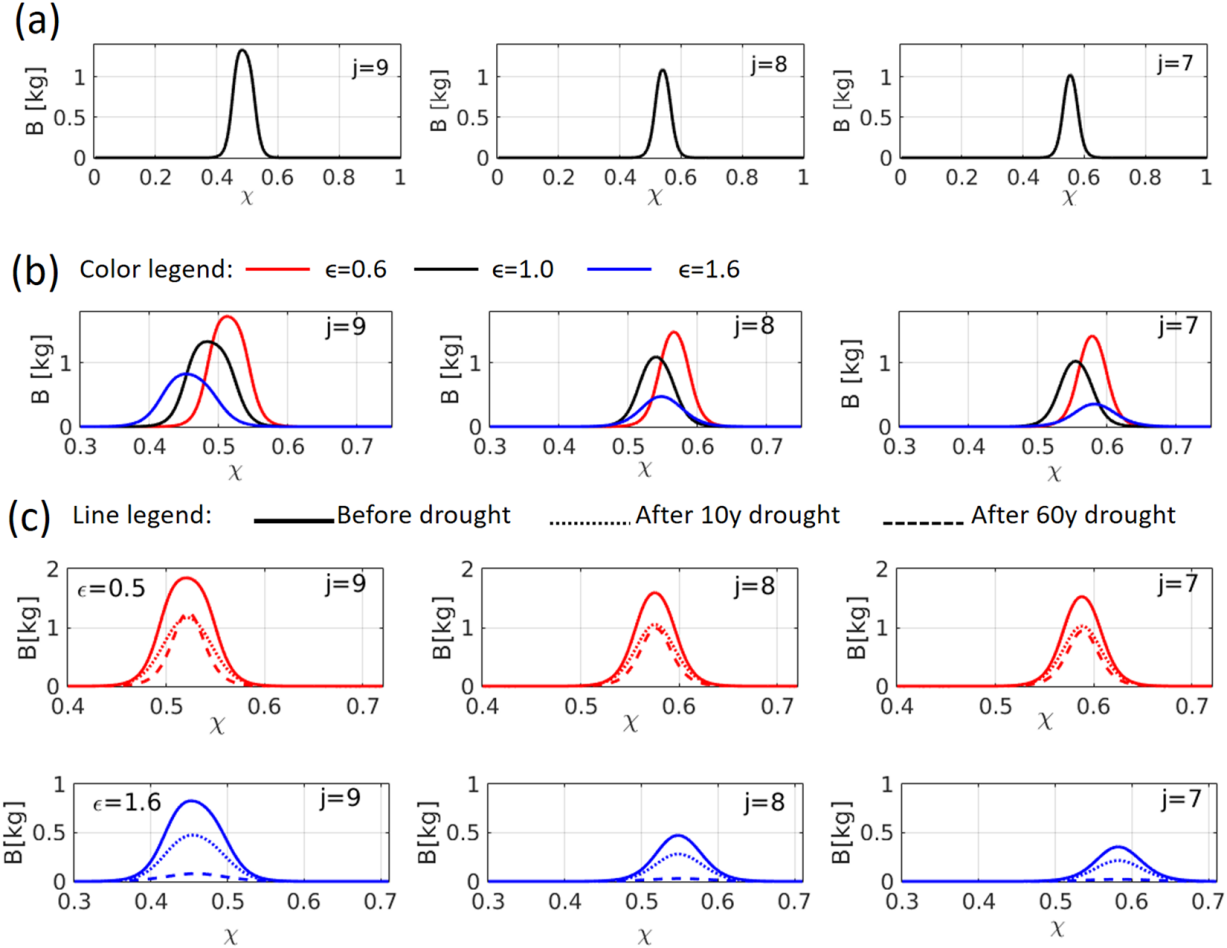
Biomass pulse solutions of Eqs. (2) along the tradeoff axis $\chi$ in the top 3 terraces of a 9-terrace riverbed. (**a**) Asymptotic solutions for a linear tradeoff $({\epsilon }=1)$. The width of the pulse (functional diversity) and the area it encloses (total biomass) decrease as we go downstream from the top terrace $(j=9)$, and the pulse position shifts to higher $\chi$, reflecting a compositional change towards higher investment in below-ground biomass at the expense of above-ground biomass. These changes become negligible below the $j=6$ terrace, indicating weak water-flow connectivity between the lower terraces. (**b**) Asymptotic solutions for a convex tradeoff (${\epsilon }=0.6$, red), linear tradeoff (${\epsilon }=1$, black) and concave tradeoff (${\epsilon }=1.6$, blue). Convex (concave) tradeoff increases (decreases) biomass but decreases (increases) functional diversity. (**c**) The riverbed response to 10 years long (dotted lines) and 60 years long (dashed lines) periods of reduced ${\rm{MAP}}$ (from 100 mm to 40 mm), for a convex (${\epsilon }=0.6$, red) and concave (${\epsilon }=1.6$, blue) tradeoffs. The solutions show severe biomass decline with respect to the original values (solid lines) for a concave tradeoff as compared with a convex one. Parameters are as in Table 1 and $k=6$.

Integrating the model equations for sufficiently long times we obtained asymptotic biomass distributions, as Fig. 4a shows, for a riverbed consisting of $N=9$ terraces with a linear tradeoff (${\epsilon }=1$). The asymptotic pulse-shape distributions contain information about three community-level properties: the total biomass (pulse area), functional diversity (pulse width) and community composition (pulse position)^20^. Inspecting the distributions, from the uppermost terrace $j=9$ to terraces downstream, we reveal declines in total biomass and functional diversity, which occur mostly across the first three terraces, and a composition shift to functional groups that invest more in below-ground biomass (higher $\chi$).

### Effects of nonlinear tradeoffs

The general trends along the riverbed shown in Fig. 4a for a linear tradeoff $({\epsilon }=1)$, that is, decline in total biomass and functional diversity and composition shift, also hold for nonlinear tradeoffs, concave $({\epsilon } > 1)$ and convex $({\epsilon } < 1)$, as Fig. 4b shows for the three uppermost terraces (results for the lower terraces are not shown as the variations become negligibly small). The nonlinearity, however, introduces two additional effects. As the tradeoff changes from convex to concave the change in community composition (pulse position) along the riverbed increases and the total biomass decreases. This is in line with the view of a concave tradeoff as representing a pool with a wide range of equally competitive functional groups that are farther away (in trait space) from the ideal functional group (red circle in Fig. 2), as compared to a convex pool.

The composition change of the community along the terraces implies higher functional diversity of the whole terraced riverbed as compared with any individual terrace. This effect is particularly significant for concave pools for which the composition change downstream is larger.

Thus, riverbeds with concave pools of functional groups are expected to show a significantly higher functional diversity, in comparison to convex pools, but lower total biomass.

### Response to droughts

The lower biomass associated with concave pools may bear on the response of the community to droughts. Figure 4c shows the responses to 10-years long and 60-years long periods of dry climate (MAP reduction from 100 mm/y to 40 mm/y) applied to concave and convex pools. While convex pools show a moderate biomass decline for both periods of dry climate, concave pools show a severe decline for the longer dry period. Continuation of the model simulations for a 10 years period of normal climate (100 mm/y) after the 60 years of dry climate show very fast recovery for convex pool (100% of the original biomass) and very slow recovery (8.4% of the original biomass) for concave pool.

**Figure 5:**
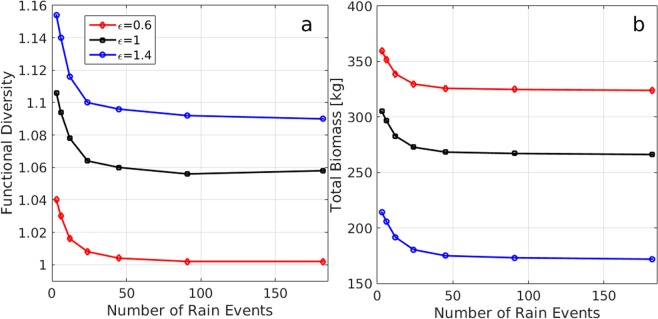
Effects of rainfall regimes on functional diversity and total biomass for the entire riverbed (9 terraces) for convex $({\epsilon } < 1)$, linear $({\epsilon }=1)$, and concave $({\epsilon } > 1)$ tradeoffs. Functional diversity (**a**) and total biomass (**b**) both decrease as the number of rain events increase (keeping MAP constant). For a given number of rain events functional diversity decreases (**a**) while total biomass increases (**b**) as the tradeoff changes from concave (blue) to convex (red).

Changing the number of rainfall events keeping MAP constant may have strong effects on functional diversity and total biomass, as Fig. 5 shows. The functional diversity and total biomass calculated for the entire riverbed sharply decline as the number of rain events increases and their intensity decreases, and approach constant values as the rainfall drops to levels for which runoff contributions from the surrounding hillslopes of the terrace become negligible. These behaviors hold for all pool types, convex, linear and concave. Comparing the functional diversity and total biomass for these pool types, at any given number of rain events, we recover the opposite trends that functional diversity and total biomass follow when the tradeoff-nonlinearity ${\epsilon }$ is changed, as discussed earlier.

## Discussion

Studies of terraced riverbeds have focused mainly on their function as agricultural systems and on the rehabilitation and conservation of abandoned riverbeds to prevent soil erosion and fertility loss^29^. Only a few field studies have focused on the ecological aspects of plant colonization in abandoned terraced riverbeds and the novel ecosystems this colonization has created^30^. In this paper we used a mathematical-modeling approach to study factors that regulate these novel ecosystems and affect ecosystem attributes such as productivity and diversity. We applied a meta-ecosystem modeling approach^21,22^ to earlier dryland-ecosystem models^24^, to obtain a model for a terraced riverbed that captures the relationships between rainfall pattern, runoff coupling between adjacent terraces, and the differential plant-community responses in the terraces in terms of biomass production and functional diversity. Our model studies show that rainfall patterns and functional properties of species pools regulate biomass production, functional diversity, and responses to extreme droughts, at the levels of a single terrace and whole riverbed, as discussed below^31^.

Based on our model studies, rainfall and its redistribution by runoff are the main drivers of ecosystem development in terraced dry riverbeds. The rates of the cascading process of water flow, rainfall → runoff → soil moisture → vegetation, are regulated by the rainfall pattern, i.e. by the temporal distribution of rainfall events in terms of frequency and magnitude. Varying the number of rainfall events in a year, keeping the mean annual rainfall (MAP) constant, we found that a large number of weak rainfall events results in low total biomass and low functional diversity (Fig. 5). We attribute these outcomes to the negligible hill-slope runoff that is generated by weak rainfall events. The low total biomass is a consequence of the low soil-water content in the absence of runoff contributions. The low functional diversity is a consequence of the high similarity among terraces; as the main water resource in all terraces is direct rainfall, their community structure is similar too. By contrast, in the case of a few strong rainfall events, the generated runoff provides an additional source of water that increases not only the total biomass but also the functional diversity. This is because of the decreasing runoff contributions to terraces down the riverbed, and the consequent different communities that establish there: functional groups specializing in capturing light (low $\chi$) in the water-rich upper terraces vs. functional groups specializing in capturing soil-water (high $\chi$) in the water-poor lower terraces (Fig. 4). The functional diversity at the riverbed level takes into account the low $\,\chi$ functional groups in the uppermost terrace and the high $\chi$ functional groups in the lowermost terrace. It is, therefore, necessarily higher than the functional diversity in the case of many weak rainfall events, where similar communities establish in all terraces. We thus conclude that fewer stronger rainfall events result in more functional ecosystems.

We are not aware of any experimental study aiming at testing directly the relationship between rainfall pattern and properties of properties of terraced-riverbed ecosystems. However, some information about this relationship can be inferred from studies of human-made systems termed “Limans” in the central Negev, which can be regarded as systems consisting of a single terrace per watershed^32–34^. In particular, it has been found^35^ that most annual rainfall events are in low magnitude and high frequency, and that the low-magnitude high-frequency events redistribute the rain within the slopes of the drainage area only. Runoff contributions to the riverbed occur only during rare high-magnitude events. Thus, rainfall events regulate ecological responses, such as species diversity, mostly on slopes, while the regulation within the riverbed occurs only during rare intense rainfall events. These results are in line with the model predictions shown in Fig. 5, namely, that biomass production and functional diversity are hardly affected by the rainfall pattern in the high-frequency range, but show substantial changes in the low-frequency range.

We adopted in this study a trait-based modeling approach, which directly bears on functional diversity and ecosystem function^36–38^. The approach allowed us to study the filtering of species traits from a set of species pools and the local community structures that emerge from interspecific competition for water and light under various rainfall regimes. The species pools we consider consist of functional groups that make different tradeoffs between two resource acquisition strategies: investment in above-ground biomass to capture canopy resources, and investment in below-ground biomass to capture soil resources. Two major forms of such tradeoffs, and thus of species pools, can be distinguished according to their shapes in trait plane: convex tradeoffs (${\epsilon } < 1$ in Fig. 2) and concave tradeoffs (${\epsilon } > 1$ in Fig. 2). Empirical information about tradeoff shapes in plant ecology is very limited^39^, but a recent study provides insightful information for the particular tradeoff we consider here^40^. This study focused on specific leaf area (SLA) and specific root length (SRL) as functional traits associated with above-ground and below-ground resource capture, and while graphs of SLA vs. SRL suggest both positive and negative relations between the two functional traits, replotting the data as graphs of SLA vs. SRL/SLA, to better correspond to the model’s functional traits $K$ and $E$, results in a clear concave tradeoff, (see Fig. A2 SM). This result supports the tradeoff relation (1) that we assume between $K$ and $E$, and points towards the possible existence of concave tradeoffs in dryland species pools.

As pointed out earlier, a heuristic view of the difference between convex and concave pools is provided in terms of the distance $s(\chi )$ in the $E,K$ trait plane between a functional group represented by a point $\chi$ on the tradeoff curve and the “ideal functional group” $({E}_{max},{K}_{max})$, which does not suffer from making a tradeoff [see Eq. (1c) and red dot in Fig. 2]. Short distances imply superior environmental-filtering and competitive capabilities, which suggests that convex pools provide advantages to small sets of intermediate functional groups whose distances to the ideal functional group are significantly shorter than those of the extremal groups (i.e. near $\chi =0$ and $\chi =1$). By contrast, moderately concave pools give rise to higher similarity among functional groups, as the distance $s(\chi )$ of intermediate groups is now comparable to that of the extremal groups. As a consequence, we may expect functional diversity to increase and environmental filtering to decrease as compared to convex pools.

These expectations and their implied consequences for ecosystem function are supported by our model results. As Fig. 5 shows, concave pools give rise to higher functional diversity (Fig. 5a) and lower biomass production (Fig. 5b) as compared with convex pools, for all rainfall patterns considered. These trends appear already at the single terrace level (Fig. 4b), but are more pronounced at the whole riverbed level because of the decreasing runoff contributions to lower terraces and the consequent shift in community structure to functional groups of higher $\chi$. The shift itself increases the whole-riverbed functional diversity, and the specific shift to functional groups that invest less in biomass production contributes to lower productivity. The better performance of convex pools in terms of biomass production is also reflected in the response to periods of dry climate. As Fig. 4c shows, a long period (60 y) of reduced MAP has a moderate effect for a convex pool but dramatically reduces the biomass in the case of a concave pool. In addition, the recovery time of a concave pool, when the original MAP is resumed, is much longer than that of a convex pool.

We have studied here moderately nonlinear tradeoff curves, focusing on the difference between convex and concave pools. For such tradeoffs we found intermediate community structures that exclude functional groups with extreme investment either in shoot or in root (Fig. 4). However, tradeoffs in practice may assume stronger nonlinear forms. Consider, in particular, a highly concave tradeoff, as shown in Fig. 1 with ${\epsilon }$=4 or as the empirical data in Fig. A2 of the SM shows. The simple heuristic reasoning based on the distance function $s(\chi )$ suggests, in this case, the possible split of the community into two extreme sub-communities. The first sub-community consists of functional groups with extremely low investment in root ($E\approx {E}_{min}$) and variable investment in shoot, whereas the second sub-community consists of functional groups with extremely low investment in shoot ($K\approx {K}_{min}$) and variable investment in root. The former may be expected to dominate at relatively high MAP, whereas the latter at a relatively low MAP. At intermediate MAP, bistability of the two sub-communities may possibly exist, implying abrupt shifts in community structure as MAP decreases or increases.

Prevailing trait-based frameworks^38^ focus primarily on community assembly from a fixed species pool, paying little attention to species pools that differ in the nonlinear forms of the tradeoffs that characterize them. Our results show that convex and concave tradeoffs result in significantly different community-level properties. These essential differences call for additional theoretical and empirical studies of convex vs. concave pools, in terraced riverbeds and in other dryland meta-ecosystems, and their implications to functional diversity, biomass production, resilience to droughts, and ecosystem management. To this end, the modeling approach pursued here in the context of terraced riverbeds can be modified or extended in several directions, including the consideration of additional tradeoffs, such as fast growth versus ability to withstand a resource stress^41^, and the modification of the water-connectivity architecture.

Hydro-geo-eco legacies shape the long-term structure, composition, and function of terraced systems. Using a model that captures essential aspects of these legacies we uncovered basic determinants of plant-community structure, such as the relation between number and intensity of rainfall events, and the nonlinear forms of tradeoff relations. Mechanistic insights obtainable in this way, can increase our scientific-based knowledge for effective management of terraced riverbeds. We suggest that various ecosystem services, such as regulation of water flow and primary production, can be achieved by re-maintenance of these cultural landscapes according to these insights.

## Methods

We study the model Eq. (2) numerically using precipitation regimes that mimic a few essential aspects of typical intra-annual rainfall patterns in the Sde-Boker area of the Negev Desert, Israel. That pattern, shown in Fig. A1a in the Supplementary Material (SM), is characterized by a dry summer and singular rainfall events during the winter, adding up to a mean annual precipitation (MAP) slightly less than 100 mm. We simplify the actual rainfall regime by considering periodic precipitation regimes with annual periodicity, $P(t+1)=P(t)$, and an intra-annual square-wave pattern of the form

5 $$P(t)=A[1-\theta (t-1/2)]\mathop{\sum }\limits_{i=1}^{k}[\theta (t-{t}_{2i-1})-\theta (t-{t}_{2i})],\,0\le t\le 1,$$

where $$k$$ is the number of rainfall events occurring at times $${t}_{1},\,\ldots ,{t}_{k}$$ limited to the first half year, $${t}_{2i}-{t}_{2i-1}=T$$ is the equal duration of any rainfall event, $$A$$ is the rainfall intensity and $$\theta (t)$$ is the unit step function [$$\theta (x)=0\,{\rm{for}}\,x < 0,\,\theta (x)=1\,{\rm{for}}\,x\ge 0]$$. An example for such a rainfall regime is given in Fig. A1b in the (SM). We vary the number of rainfall events ($$k)$$ and their intensity ($$A)$$ keeping the product $$kAT$$ constant and equal to $${\rm{MAP}}=100[{\rm{mm}}]$$. This choice allows us to compare the responses of terraced riverbeds to precipitation regimes ranging from many weak rainfall events to a few strong events.

The response of a terraced riverbed to a particular rainfall regime is studied by calculating the year-end values of the biomass variables, ${B}_{ij}={B}_{j}({\chi }_{i})$, at long times, from which the biomass distributions ${B}_{j}(\chi )$ along the tradeoff axis $\chi$ for all terraces $j=1,\ldots ,N$, are constructed.

The model equations were solved numerically using forth order Runge-Kutta method. The number of species (m) was 500 and the system included 9 terraces. The daily precipitation was given as an input file according to Eq. (5). The simulations were integrated to sufficiently long times to ensure convergence to steady-state solutions. Numerical values for the model parameters used in these simulations are displayed in Table 1.

## Supplementary information

Supplementary information

## Data Availability

There is no data available to this paper since it presents a theoretical work.
