# Supplementary material for: A model study of terraced riverbeds as novel ecosystems
Source: Sci Rep. 2020 Mar 2;10:3782. doi: 10.1038/s41598-020-60706-y (PMC7052233; doi:10.1038/s41598-020-60706-y)
Supplement: Supplementary file 1 — Supplementary information [file 41598_2020_60706_MOESM1_ESM.pdf]

Supplementary material for:

A model study of terraced riverbeds as novel ecosystems

Hezi Yizhaq[1], Moshe Shachak[2], Ehud Meron[1,3]

[1]Department of Solar Energy and Environmental Physics, Blaustein Institutes for Desert Research, Ben-Gurion University of the Negev, Sede Boker Campus, Beer Sheva 84990, Israel

[2]Mitrani Department of Desert Ecology, Blaustein Institutes for Desert Research, Ben-Gurion University of the Negev, Sede Boqer Campus 8499000, Israel

[3]Physics Department, Ben-Gurion University of the Negev, Beer Sheva 84105, Israel

Appendix 1

Figure A1 Annual rainfall regimes

Figure A2   Empirical tradeoff relation

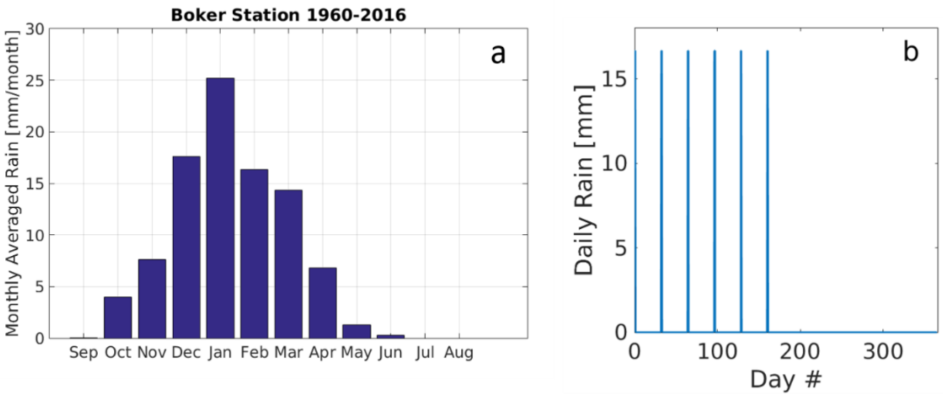

Figure A1. Annual rainfall regimes. (a) Averaged monthly rainfall distribution in Sde Boker, Negev desert, between 1960-2016, showing the confinement of the rainy season to six consecutive months (excluding months below 5mm). The mean annual precipitation is 93.5 mm. (b) Rainfall regime, calculated from Eq. 4, for 6 rain events, each a single day long, accumulating to $\mathrm{MAP} = 100[\mathrm{mm}]$.

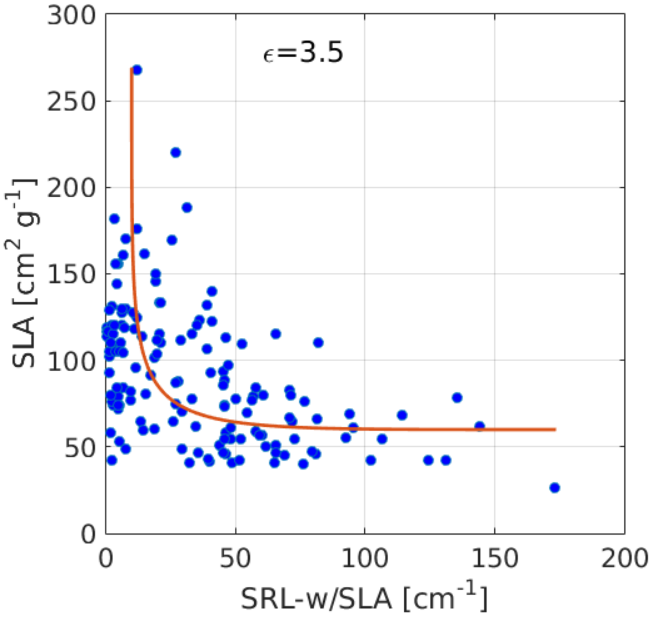

Figure A2. Empirical tradeoff relation between specific leaf area (SLA) and specific whole-root length (SRL-w) per leaf area. The circles denote data that has been made available by Cheng et al. (2016). The red line represents a best fit to the data using Eq. (1) with $\epsilon = 3.5$.
